# Supplementary material for: Influence of Ceria Addition on Crystallization Behavior and Properties of Mesoporous Bioactive Glasses in the SiO2–CaO–P2O5–CeO2 System
Source: Gels. 2022 May 31;8(6):344. doi: 10.3390/gels8060344 (PMC9222617; doi:10.3390/gels8060344)
Supplement: Supplementary file 1 [file gels-08-00344-s001.zip › gels-1728283-supplementary.pdf]

Supplementary Information

Influence of ceria addition on crystallization behavior and properties of several bioactive glasses in the SiO_2–CaO-P_2O_5-CeO_2 system

Elena Maria Anghel, Simona Petrescu *, Oana Catalina Mocioiu, Jeanina Pandele Cusu and Irina Atkinson*

Institute of Physical Chemistry 'Ilie Murgulescu" of Romanian Academy, Spl. Independentei 202, Spl. Independentei 202, 060021, Bucharest, Romania

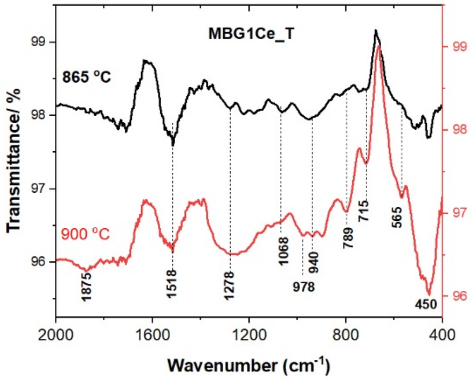

Figure S1. FT-IR spectra of the MBG1Ce annealed at 865 and 900 °C

Table S1UV-Raman band positions of the annealed MBG5Ce_T spectra and assignments within 330-1200 cm^{-1} spectral range

CeO_2	MBG5Ce_T				Assignment	Reference
	RT	830	876	906		
		392	390	404	Si-O-Si bending	
		437			[δ(O^--P-O^-)] in Q^0(P)	
456	459		446		F_{2g} modes of CeO_2	43
				464	quartz	44
	531	499	549	512		
	575	562		572	Si-O-Si vibrations in Q^2(Si)	
	620	595, 624	590		$CePO_4$ and Si-O-Si	45

					vibrations in $Q^2(Si)$	
			642	634	Si-O-Si bending in Wollastonite	46
	833		827	785	P-O-P symmetric stretch	46
	848	848	857	840	Si-O stretch of $Q^0(Si)$	24
	947	937		944	P-O stretch of $Q^0(P)$	24 and 43
	974	990	950	967	Si-O stretch in $Q^2(Si)$	24
		1097			$\nu_1(CO_3^{2-})$	47
	1152			1153	Si-O-P bonds (1140 cm^{-1})	32
1167			1186		2LO modes of ceria, $\nu_3(PO_4)$ and Si-O stretch of $Q^4(Si)$	44, and 24
	1237		1272	1249		
	0.9981	0.9990	0.9981	0.9989	R^2	

Table S2 Results (T_x and T_{c1}) of fitted DSC curves of the MBG(0/1/5Ce)

β /°C min.$^{-1}$	**MBG0Ce**				**MBG1Ce**				**MBG5Ce**			
	**T_x /°C**	**T_p /°C**	**FWHM /°C**	**Tx-Tg /°C**	**Tx /°C**	**Tp /°C**	**FWHM /°C**	**Tx-Tg /°C**	**Tx /°C**	**Tp /°C**	**FWHM /°C**	**T_x-T_g /°C**
5	859	868	21.98	345	854	855	18.75	214	761	815	48.66	123
10	866	878	23.58	340	853	865	19.65	211	788	830	41.17	149
15	867	882	24.72	344	860	872	21.40	208	794	837	41.08	145
20	871	888	26.05	343	861	875	21.41	206	800	842	40.13	150
25	876	892	27.35	348	860	878	23.11	201	811	845	38.71	159

Tx=onset temperature of the first crystallization event

References:

[24] H. Aguiar, J. Serra, P. González, B. León, Structural study of sol–gel silicate glasses by IR and Raman spectroscopies, J. Non-Cryst. Solid. 355 (2009) 475–480. doi:10.1016/j.jnoncrysol.2009.01.01

[30] M.H. Brooker, Raman and i.r. spectra of zinc, cadmium and calcium nitrate: a study of the low temperature phase transitions in calcium nitrate, Spectrochim. Acta 1975; 32A: 369-377.Doi:10.1016/0584-8539(76)80090-6

[43] Padilla, S.; Roman, J.; Carenas, A.; Vallet-Regi, M. The influence of the phosphorus content on the bioactivity of sol–gel glass ceramics, Biomaterials 2005; 26: 475–483. doi:10.1016/j.biomaterials.2004.02.054

[44] M. M. Pereira, A. E. Clark, L. L. Hench, Calcium phosphate formation on sol-gel-derived bioactive glasses in vitro, J. Biomed. Mater. Res. 1994; 28: 693-698. Doi: 10.1002/jbm.820280606

[45] De Ligny, D.; Neuville, D.R. 12-Raman spectroscopy: a valuable tool to improve our understanding of nucleation and growth mechanism. in From Glass to Crystal. Nucleation, growth and phase separation: from research to applications. Neuville, D.R.; Cormier, L.; Caurant, D.; Montagne L. eds. EDP Sciences 2017, Les Ulis, France, pp 319-344.

[46] Taniguchi, T.; Watanabe, T.; Sugiyama, N.; Subramani, A.K.; Wagata, H.; Matsushita, N.; Yoshimura, M. Identifying defects in ceria-based nanocrystals by UV resonance Raman spectroscopy. *J. Phys. Chem. C***2009**, *113*, 19789–19793. Doi:10.1021/jp9049457

[47] Olivares, M.; Zuluaga, M.C.; Ortega, L.A.; Murelaga, X.; Alonso-Olazabal, A.; Urteaga, M.; Amundaray, L.; Alonso-Martina, I.; Etxebarria, N. Characterisation of fine wall and eggshell Roman pottery by Raman spectroscopy. *J. Ram. Spectrosc.***2010**, 41, 1543–1549. doi: 10.1002/jrs.2748
